# Increased H3K4me3 methylation and decreased miR-7113-5p expression lead to enhanced Wnt/β-catenin signaling in immune cells from PTSD patients leading to inflammatory phenotype

**DOI:** 10.1186/s10020-020-00238-3

**Published:** 2020-11-14

**Authors:** Marpe Bam, Xiaoming Yang, Brandon P. Busbee, Allison E. Aiello, Monica Uddin, Jay P. Ginsberg, Sandro Galea, Prakash S. Nagarkatti, Mitzi Nagarkatti

**Affiliations:** 1 Department of Pathology, Microbiology and Immunology, School of Medicine, University of South Carolina, Columbia, SC 29209 USA; 2 William Jennings Bryan Dorn Veterans Medical Center, 6439 Garners Ferry Road, Columbia, SC 29209-1639 USA; 3 Department of Epidemiology, UNC Gillings School of Global Public Health, University of North Carolina, Mcgavran-Greenberg Hall, Chapel Hill, NC 27599-7435 USA; 4 Genomics Program, University of South Florida College of Public Health, 3720 Spectrum Blvd., Tampa, FL USA; 5 School of Public Health, Boston University, 715 Albany Street-Talbot 301, Boston, MA 02118 USA

## Abstract

**Background:**

Posttraumatic stress disorder (PTSD) is a psychiatric disorder accompanied by chronic peripheral inflammation. What triggers inflammation in PTSD is currently unclear. In the present study, we identified potential defects in signaling pathways in peripheral blood mononuclear cells (PBMCs) from individuals with PTSD.

**Methods:**

RNAseq (5 samples each for controls and PTSD), ChIPseq (5 samples each) and miRNA array (6 samples each) were used in combination with bioinformatics tools to identify dysregulated genes in PBMCs. Real time qRT-PCR (24 samples each) and in vitro assays were employed to validate our primary findings and hypothesis.

**Results:**

By RNA-seq analysis of PBMCs, we found that Wnt signaling pathway was upregulated in PTSD when compared to normal controls. Specifically, we found increased expression of *WNT10B* in the PTSD group when compared to controls. Our findings were confirmed using NCBI’s GEO database involving a larger sample size. Additionally, in vitro activation studies revealed that activated but not naïve PBMCs from control individuals expressed more *IFN*γ in the presence of recombinant *WNT10B* suggesting that Wnt signaling played a crucial role in exacerbating inflammation. Next, we investigated the mechanism of induction of *WNT10B* and found that increased expression of *WNT10B* may result from epigenetic modulation involving downregulation of hsa-miR-7113-5p which targeted *WNT10B*. Furthermore, we also observed that *WNT10B* overexpression was linked to higher expression of H3K4me3 histone modification around the promotor of *WNT10B*. Additionally, knockdown of histone demethylase specific to H3K4me3, using siRNA, led to increased expression of *WNT10B* providing conclusive evidence that H3K4me3 indeed controlled *WNT10B* expression.

**Conclusions:**

In summary, our data demonstrate for the first time that Wnt signaling pathway is upregulated in PBMCs of PTSD patients resulting from epigenetic changes involving microRNA dysregulation and histone modifications, which in turn may promote the inflammatory phenotype in such cells.

## Introduction

Exposure to traumatic life events can lead to development of Post-traumatic stress disorder (PTSD) (American Psychiatric Association 2013). PTSD is prevalent among victims of traumatic events, assaultive violence and domestic abuse, as well as in War Veterans. An approximate 3.6% of American adults between 18 and 54 years, about 30% of Vietnam War Veterans (Kessler et al. 2005), 8% of Gulf War Veterans (Ramchand et al. 2010) have been detected with PTSD. Additionally, up to 40% of returning combat Veterans from Iraq and Afghanistan (Operation Enduring Freedom and Operation Iraqi Freedom) have been estimated to develop PTSD (Hoge and Warner 2014). Prevalence of lifetime PTSD among civilians in a stressful urban environment has recently been reported to be 17.1% in the Detroit Neighborhood Health Study (DNHS) (Goldmann et al. 2011). PTSD affects social and professional functioning, emotional well-being, along with physical health and health-related quality of life. In addition to a heterogeneous array of symptoms of intrusive thoughts, avoidance and arousal behaviors, and negative cognitions (American Psychiatric Association 2013), patients with PTSD may also experience increased risk of cardiovascular disease, progressive decrease in muscle bulk, osteoporosis and fractures, cancer, arthritis, digestive disorders (Boscarino 2004; O'Donovan et al. 2015; Brudey et al. 2015). Although the precise mechanisms of these clinical disorders in PTSD patients have not been fully elucidated, chronic inflammation has been implicated in these diseases (Daskalakis et al. 2016; Speer et al. 2018).

Recently, there have been many reports on immune dysfunction in PTSD. Increased numbers of inflammatory immune cells were reported in abused women with PTSD symptoms (Lemieux et al. 2008), as well as in male veterans with PTSD (Bersani et al. 2016). We reported previously that in PTSD patients, there is significant increase in peripheral blood mononuclear cells (PBMCs), the number of CD4+, CD8+, NK and B cells in PBMC, and production of pro-inflammatory cytokines such as IFNγ, IL-17, RANTES and IL-12 (Zhou et al. 2014; Bam et al. 2016a). Moreover, we reported dysregulation of immune system gene expression in PTSD by RNA-Seq and provided evidence suggesting altered miRNA and DNA methylation could be mechanisms contributing to the dysregulated gene expression (Bam et al. 2016b). At present, the general consensus has been that PTSD patients experience chronic inflammation severely affecting quality of life (Daskalakis et al. 2016; Speer et al. 2018; Zhou et al. 2014; Bam et al. 2016a; Gola et al. 2013; Michopoulos et al. 2015). Thus, understanding the origin of chronic inflammation may help address the comorbidity associated with PTSD. Despite the observation of chronic inflammation in PTSD patients, the molecular mechanisms underlying dysregulation of the immune system are in need of further clarification.

Wnt/β-catenin signaling pathway is known to regulate cellular proliferation, differentiation, polarization and cell fate (Logan and Nusse 2004; Nusse 2005). The Wnt signaling pathway has been implicated in certain inflammatory diseases, such as rheumatoid arthritis (Miao et al. 2013), chronic ulcerative colitis (Rogler 2014), periodontitis (Galli et al. 2011), lung diseases including asthma and chronic obstructive pulmonary disease (Skronska-Wasek et al. 2018), but the precise mechanisms remain to be characterized. It was also reported that *WNT5A* induces cyclooxygenase-2 expression and enhances inflammatory cytokines rapidly (Kim et al. 2010). In light of the above findings on role of WNT signaling in inflammation, in the present study, we screened PTSD PBMCs for potential WNT signaling pathways that are altered, and found that there was increased expression of *WNT10B* in PTSD samples. We further characterized the epigenetic mechanisms through which WNT signaling is upregulated in PTSD and observed that WNT signaling was regulated by altered histone methylation and miRNA expression in PTSD PBMCs which in turn regulated inflammatory cytokine genes such as *IFN*γ. We believe that this is the first report which demonstrates that dysregulation of *WNT10B* can result in the upregulation of proinflammatory cytokines and may contribute to the inflammatory state in PTSD.

## Materials and methods

### Patients

All participants were provided informed and written consent to participate in the study. The study was approved by the Institutional Review Board. In the current study, we included data from 81 samples (40 controls and 41 PTSD) which made up two study datasets. In the first set (Dataset 1), we included 48 samples (24 each controls and PTSD) in total and used two groups of PTSD. One group of PTSD consisted of Veterans of either the 1991 Persian Gulf or the OEF-OIF campaigns, as described earlier (Zhou et al. 2014; Bam et al. 2016a, b), and the second group consisted of study participants from the Detroit Neighborhood Health Study (DNHS) (Uddin et al. 2010) who met diagnostic criteria for PTSD. We randomly included both genders for our study. In the Veterans group, we excluded individuals suffering from any immune related diseases, undergoing any immunotherapy, anyone under alcohol dependency or immune suppressing illegal drugs. In Table 1 we have provided the demographic of the samples. The samples from Dataset 1 were used for the RNA-seq, ChIP-seq, miRNA array and qPCR validation experiments. The second set (Dataset 2) included 33 PBMC samples obtained from 16 controls and 17 PTSD patients (GSE860) (Segman et al. 2005). We used the data from Dataset 2 to confirm our observations in a replication group. All donors of the first group (war veterans) in Dataset 1 were first clinically assessed by professionals for PTSD employing the psychometric properties of the PTSD Checklist (PCL) (Blanchard et al. 1996) and PTSD diagnosis was validated by the Clinician Administered PTSD Scale (Blake et al. 1995) according to formal diagnostic criteria in the Diagnostic and Statistical Manual of Mental Disorder (DSM-IV) (American Psychiatric Association 2013). Exclusion criteria included current alcohol and other substance abuse, undergoing immunosuppressive drug treatment, or having immunosuppressive disease. The control individuals were age-matched healthy volunteers, who did not have any symptoms of active infection or any history of immune compromise such as HIV, cancer, pregnancy or on chronic steroid therapy. DNHS participants were assessed for PTSD via structured telephone interview using the PTSD checklist (PCL) (Blanchard et al. 1996), as previously described (Uddin et al. 2010). A subset of participants for this study was also validated via in-person clinical interviews using the Clinician Administered PTSD Scale mentioned earlier (Blake et al. 1995). The demographic information of the individuals included in Dataset 1 is provided in Table 1.

**Table 1:** Demographics of the samples included for the study

Parameters	Control (n = 24)	PTSD (n = 24)	*p* value
Age (y)	47.9 (2.38)	44.31 (1.9)	0.35
Race			
AA^a^	14 (58%)	15 (62%)	
CA^b^	10 (42%)	8 (33%)	
Hispanic	0	1 (4%)	
Gender			
Male	14 (58%)	12 (50%)	
Female	10 (42%)	12 (50%)	

The values in the parentheses represents ± SEM (age) and percentage (race and gender). *p* value was obtained by performing Student’s *t* test. These samples include both the veterans and the DNHS samples

^a^African American

^b^Caucasian

### Sample collection and RNA isolation

Peripheral blood samples (2–10 ml) collected in EDTA coated collection tubes were processed to isolate PBMCs by density centrifugation using Ficoll-Paque (GE Healthcare, Uppsala, Sweden). Using a universal kit (AllPrep DNA/RNA/miRNA Universal Kit, Qiagen, Valencia, CA) recommended for simultaneous isolation of high quality DNA and total RNA, including miRNAs, all the three entities were isolated from the same PBMCs and immediately frozen at − 80 °C until use.

### RNA-sequencing (RNA-seq)

Five control and 5 PTSD patient samples from Dataset 1 were analyzed to get the gene expression profile and later the genes were validated by including 24 each samples from both groups. Libraries were constructed using Illumina TruSeq RNA Sample Preparation kit as described in Bam et al. (2016a, b). Briefly, total RNA from PBMCs was isolated using the Qiagen AllPrep DNA/RNA/miRNA Universal Kit. Following manufacturer instruction, oligo-dT beads were then added to 1 µg of total RNA to isolate mRNA. The mRNA obtained was fragmented to 200–400 bases. The RNA fragments were then reverse transcribed into double stranded cDNA fragments followed by repairing the DNA fragments to generate blunt ends using T4 DNA polymerase, Klenow polymerase and T4 polynucleotide kinase. The DNA fragments were purified using Qiagen PCR purification kit (Qiagen #28004), following which an “A” base was added to the 3′ end of the blunt DNA fragment by Klenow fragment. Using DNA ligase, sequencing adapters were ligated to the ends of DNA fragments. The libraries were then amplified by limited PCR cycles (15 cycles) using primers provided in the kit. The PCR products were gel separated by running in a 2% agarose gel and fragments with sizes ranging from 250 to 400 bp were excised and purified using the QiAquick Gel Extraction Kit (Qiagen #28704). The concentration of the libraries was determined by a NanoDrop spectrophotometer (Thermo Scientific, Wilmington, DE). The library was sequenced by Illumina HiSeq 2000 at Tufts University Genomic core facility. Raw sequencing reads (50 bp single-end) were mapped to human genome build hg19 using Tophat 2 (Kim et al. 2013). The accepted hits were used for assembling transcripts and estimating their abundance using Cufflinks. The differentially expressed gene, promoter usage and splicing forms were determined by Cuffdiff and Cuffcompare (Trapnell et al. 2010).

### Chromatin immunoprecipitation sequencing (ChIP-Seq)

Samples (6 each of control and PTSD) were obtained from the Detroit Neighborhood Health Study (DNHS) collection (Uddin et al. 2010). The present study was performed in collaboration with the DNHS study. Thus, only a fraction of the total samples from the DNHS study was included. For chromatin and library preparation from as low as 10,000 cells, we used the following kits from Diagenode (Diagenode Inc. NJ, USA): True MicroChIP & MicroPlex Library Preparation™ Package (cat# C01010131) and MicroPlex Library Preparation Kit v2 (cat# C05010014). Following the instructions of the kit manufacturer, PBMCs (20–40 × 10^3^ cells) from both controls and PTSD patients were treated with 27 µl of 36.5% formaldehyde to cross link histone and DNA. The mixture was incubated for 10 min at room temperature with gentle shaking. To quench the formaldehyde, 115 µl of 10× glycine was added and incubated further for 5 min at RT. Cells were then pelleted and washed with cold PBS for 2 times. Nuclear membrane disruption and chromatin shearing was achieved in lysis buffer solution by sonicating the samples at 4 °C in a temperature controlled Bioruptor sonicator (Diagenode Inc. NJ, USA) following manufacturer instructions. Sample was centrifuged at 13,000 rpm for 10 min and the supernatant was used for chromatin immunoprecipitation (ChIP) with antibody against human H3K4me3 from Diagenode Inc. (cat# C15410003-50). After overnight IP at 4 °C with gentle rotation, protein G beads were added and incubated further for 2 h. The protein G bead bound antibody with chromatin was separated from the solution by applying magnetic force, washed three times with low salt wash buffer and once with high salt wash buffer. Each wash lasted for 5 min with gentle rotation at 4 °C. Chromatin was re-suspended in elution buffer and the cross link was reversed by treating the immunoprecipitated chromatin with proteinase K at 65 °C for 45 min with constant vortexing. Size selection of the fragments was performed using SPRI beads (cat# B23317) from Beckman Coulter. DNA fragments of desired size was then re-suspended in water and quantified. The sequencing library was constructed using MicroPlex Library Preparation Kit v2 (cat# C05010014, Diagenode Inc.) according to the manufacturer’s instruction and sequenced by Illumina NextSeq500 at University of South Carolina School of Medicine.

### Micro-RNA microarray

We included 6 controls and 6 PTSD patients for the microarray. The samples included were from the DNHS collection. Total RNA, including mRNA, miRNA and other small RNA molecules, were isolated from PBMC samples as described previously. Total RNA samples were then used in the analysis of miRNA dysregulation by miRNA array hybridization assay using the Affymetrix miRNA-4 gene chip as described by Bam et al. (2017). Raw data was obtained as signal intensities after the array was processed in Transcriptome Analysis Console (TAC) (Affymetrix, Sunnyvale, CA). Linear fold-changes in miRNA up- or down-regulation were calculated to compare the differences of all the miRNAs expressed between PTSD patients and controls. A linear fold-change of at least ± 1.5 was used as a cut off value for the inclusion of a miRNA for further analysis.

### GEO Dataset analysis

In order to further confirm our observations from the experiments with Dataset 1, we analyzed another dataset (Dataset 2) submitted to NCBI’s GEO database. The Dataset 2 has microarray generated gene expression data from PBMCs obtained from 16 controls and 17 PTSD samples (GSE860) (Segman et al. 2005). Because this study used PBMCs, the conditions are very much similar to ours as we also used PBMCs in our study. From the dataset, we collected the gene expression values as provided in the link (GSE860).

### Cell culture

THP1 cells obtained from ATCC were cultured in complete RPMI medium containing 10% FBS, penicillin and streptomycin, HEPES buffer and 2-mercaptoethanol. The incubation condition was at 37 °C and 5% CO_2_.

### In vitro knockdown with siRNA in THP1 cells

To analyze whether the presence of H3K4me3 influenced the expression of WNT10B, we knocked down KDM5B (Lysine (K)-specific demethylase 5B) and quantified WNT10B transcripts. THP1 cells were seeded at 2 × 10^5^ cells per well in a 24 well plate. After 24 h, 5pmole of siRNA was added as a mixture in Lipofectamine RNAiMAX (Invitrogen, ThermoFisher Scientific, USA) to each well by following the instruction from the manufacturer. The cells were further cultured for 48 h and harvested for RNA isolation which was later used for qRT-PCR analysis.

### Micro-RNA mimic assay

To investigate whether hsa-miR-7113-5p regulated expression of WNT10B, we performed miRNA mimic transfection studies in THP1 cells. Mimics and inhibitors of the miRNA were obtained from Qiagen Inc. and transfected into the cells by employing Lipofectamine RNAiMAX (Invitrogen, ThermoFisher Scientific, USA). One day prior to transfection, 2 × 10^5^ THP1 cells were plated in a 24 well plate in 500 µl complete RPMI medium. Mimic, inhibitor and scrambled oligos were then added at an amount of 5pmole per well. Cells were cultured for the indicated time after transfection and harvested for RNA and protein isolation. RNA samples were used for qRT-PCR based quantification of mRNAs and miRNAs. Whole cell lysates were used for western blot analysis of miRNA target protein.

### WNT10B signaling pathway initiation

To analyze whether WNT10B could influence the expression of proinflammatory genes, we initiated the WNT signaling by adding recombinant human (rh)-WNT10B in PBMCs cultures. The PBMCs were isolated from healthy human individuals. Two million cells were cultured in 200 µl medium in a 96 well tissue culture plates as follows: First, the PBMCs were stimulated with Phorbol 12-myristate 13-acetate (PMA) (200 nM) for 6 h following which, 200 ng/ml rh-WNT10B was added. The PBMCs were harvested after 24 and 48 h and used for RNA and whole cell lysate (WCL) extraction for further analysis. The culture supernatants were also collected for ELISA to detect IFNγ. The following combinations were used as controls: (1) PBMCs + PMA without WNT10B; (2) PBMCs + WNT10B without PMA.

To see whether pre-exposure of cells to WNT10B, before activation of cells, can influence inflammatory gene expression, WNT10B (200 ng/ml) was first added in all the culture with PBMCs. After 24 h, PMA (200 nM) was added to activate the cells. The following condition was used for the control: PBMCs + WNT10B without PMA. The cells were then harvested 24 h after addition of PMA.

To test if chronic presence of WNT10B alone (without stimulation with PMA) can lead to increased expression of inflammatory genes in the PBMCs, we cultured PBMCs (2 × 10^6^ cells/well, in 200 µl, in a 96 well plate) with rh-WNT10B (200 ng/ml) for 96 h. Additional rh-WNT10B was added after every 24 h of culture. Cells were harvested every 24 h for RNA and whole cell lysate isolation. Supernatants were also collected for performing ELISA.

### Blocking WNT signaling

In order to investigate whether WNT10B was directly responsible for the upregulation of IFNγ expression, we blocked the WNT signaling pathway by using inhibitor of WNT response-1 (IWR-1, Sigma-Aldrich, St. Louis, Missouri, cat#I0161). IWR-1 inhibits WNT signaling by stabilizing AXIN1 (Chen et al. 2009; Kulak et al. 2015) and prevents its degradation. AXIN1 is an inhibitor of WNT signaling pathway (Li et al. 2012). PBMCs (2 × 10^6^) from healthy controls were cultured overnight (~ 18 h) with 10 µM of IWR-1 in separate wells of a 96 well culture plate in 200 µl complete RPMI medium. The following day, PMA and IWR-1 (in a 10 µl volume) were added to the culture to reach a final concentration of 200 nM PMA and 10 µM IWR-1, respectively. After 6 h, WNT10B was added to the designated wells. Cells were harvested after 24 and 48 h of addition of WNT10B for total RNA extraction. Culture supernatant was collected for ELISA.

### ELISA and Western Blot

ELISA for human IFNγ was performed on culture supernatants. The kit for ELISA was purchased from Biolegend Inc. (CA, USA) and assay was performed by following the manufacturer instructions. Reading for OD was performed in a Victor®™, 1420 Multilabel Counter (Perkin Elmer, USA) plate reader.

For the Western blot, anti-human-WNT10B was purchased from SIGMA-ALDRICH (cat#PRS4619). Whole cell lysate from THP1 cells (from 4 wells of a 24 well plate) after transfection of mimic for hsa-miR7113-5p or scramble was collected after 72 h in a 300 µl volume of M-PER solution (ThermoFisher Scientific Inc., USA) and processed as per protocol provided by manufacturer. Total protein was quantified by Qubit (ThermoFisher Scientific Inc., USA) and loaded equal amount in the SDS-PAGE. After SDS-PAGE, transfer of protein to nitrocellulose membrane was performed in an iBlot2 instrument (ThermoFisher Scientific Inc., USA). The primary antibody was used at 3 μg/ml of blocking agent solution (5% skimmed milk powder, BioRad Inc., USA) and incubated overnight at 4 °C. Secondary antibody (Cell Signaling, cat#7074S) against rabbit IgG was used at 1:2000 dilution and incubated for 1 h at room temperature. Protein bands were detected in a LI-COR blot detection system (Li-Cor Inc, Nebraska, USA).

### Statistical analyses

Data are presented as mean ± standard deviation, with each test being repeated at least three times. We performed Student’s *t* test and p < 0.05 was considered to show significant difference between two groups under consideration. The *p* values were calculated by including data from three or more experiments.

## Results

### PTSD is associated with dysregulation of immune system related genes in the PBMCs

To gain insights into whether the immune system related genes are dysregulated in PTSD patients, we performed RNA-seq screening analysis using total RNA obtained from PBMCs from healthy controls and PTSD patients, using an initial sample size of n = 5 in each group (as described previously in Bam et al. (2016a, b), followed by a larger sample size to validate the genes of interest (n = 24 in each group). Only genes with significant (p < 0.05), FPKM (Fragments Per Kilobase of transcript per Million mapped reads) value of at least 1 in one group and a log fold change of at least 0.5 were included in the final list for further analysis. We identified 326 genes that matched our criteria. In the present study, we used the same dataset published previously (Bam et al. 2016a, b) as this study is a continuation about understanding the mechanism of immune dysregulation in PTSD patients.

### Wnt/β-catenin signaling pathway genes are differentially expressed in PTSD

**Fig. 1:**
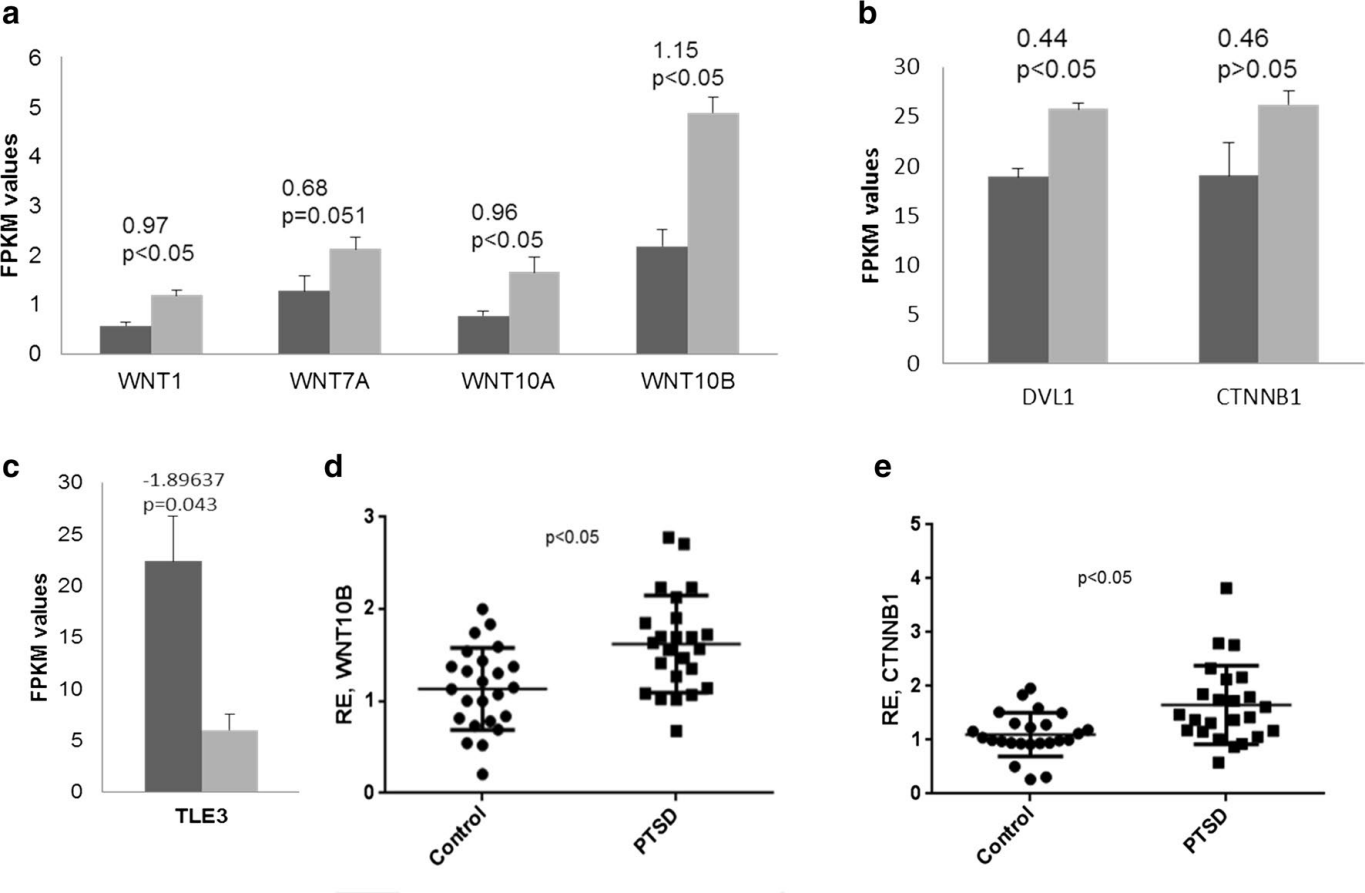
RNA-seq analysis using PBMCs from PTSD and control participants. Five control and 5 PTSD patient samples each were included for the analysis using total RNA from PBMCs as described previously by us (Zhou et al. 2014). The expression levels of different genes including WNT ligands (**a**) and WNT signaling pathway (**b**), shown as FPKM values. Black and grey bars represent control and PTSD samples, respectively. The values above the bars indicate log2 fold change as obtained after RNAseq data analysis. **c** Expression level of *TLE3* in control and PTSD patients. The transcript level of *WNT10B* and *CTNNB1was* validated by qRT-PCR in 24 control and PTSD samples each (**d**, **e**). The *p* values were derived from the RNA-seq data analysis tool (**a**–**c**) and Student’s *t* test (**d**, **e**)

Because we have observed previously (Lemieux et al. 2008) that the total cellularity of PBMCs (particularly CD4+ and CD8+ T cells) in PTSD patients was increased, we examined the expression of genes involved in cellular functions including proliferation, differentiation, polarization and activation. Interestingly, we observed that genes of the Wnt/β-catenin signaling pathway were significantly upregulated in the PBMCs of PTSD patients. Specifically, we observed upregulation of WNT ligands including *WNT10B*, *WNT10A* and *WNT7A* (Fig. 1a). Moreover, there were significantly higher transcript levels of *DVL1* (Dishevelled 1) and *CTNNB1* (Catenin Beta 1) (Fig. 1b), all of which are genes having roles downstream of the binding of WNT ligands to FZD and LRP5/6 receptors. DVL1 binds to the cytoplasmic C-terminus of FZD receptor family members and transduce the Wnt signal to downstream effectors. CTNNB1 accumulates in the cytoplasm upon binding of WNT ligand to the FZD and LRP5/6 receptors. Then, they translocate to the nucleus and act as co-activators of TCF/LEF family, which leads to activation of Wnt responsive genes. To further support our observation on Wnt/β-catenin signaling upregulation, we checked the expression of genes that inhibit this pathway. In this regard, we observed down regulation of *TLE3* (Transducin like enhancer of split 3) in PTSD patients (Fig. 1c). *TLE3* is among the ones that plays a significant role in the inhibition of the Wnt/β-catenin signaling pathway by acting as a transcriptional repressor of β-catenin/TCF4 complex (Villanueva et al. 2011; Liu et al. 2018). To support our RNA-seq data, we performed qRT-PCR validation of *WNT10B* and *CTNNB1* using a larger sample size consisting of 24 each of control and PTSD samples and observed that these genes were significantly upregulated in PTSD when compared to controls (Fig. 1d, e).

### Higher H3K4me3 signals present in PTSD around the promoters of Wnt/β-catenin signaling pathway genes

**Fig. 2:**
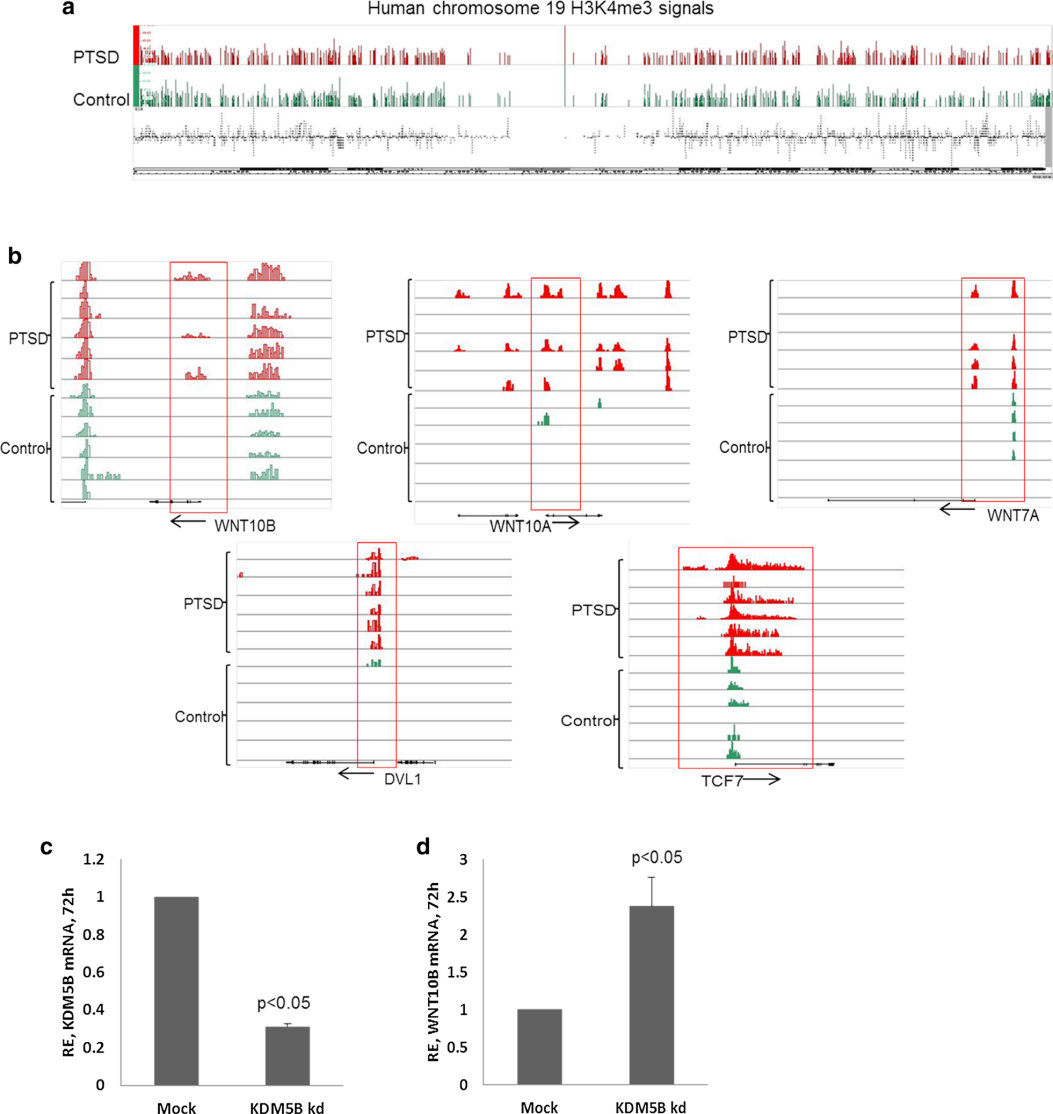
ChIP-seq analysis for H3K4me3 marks. Six PTSD and 6 control samples were analyzed. In panel (**a**), a representative H3K4me3 ChIP-seq signal obtained after visualization of the sequencing data in Integrated Genome Browser (IGB) for human chromosome 19 is shown. **b** H3K4me3 signal around 5 genes of the WNT signaling pathway in 6 controls and 6 PTSD samples. One horizontal line represents a sample. **c** KDM5B was knocked down by siRNA in THP1 cells and confirmed by qRT-PCR after 72 h. The bar graph shows level of KDM5B transcripts in siRNA transfected cells. **d** WNT10B transcript level in KDM5B knocked down cells after 72 h. (*RE* relative expression)

In order to understand the regulation of genes of the Wnt/β-catenin signaling pathway through epigenetic mechanisms, we investigated the H3K4me3 methylation pattern in PTSD and compared that with controls. We performed H3K4me3 ChIP sequencing in 6 each of control and PTSD PBMC samples. The sequencing data was visualized in Integrated Genome Browser (IGB) (Freese et al. 2016) for signals around genes of interest. Overall, at the genome level, there was no major difference in the signals between controls and PTSD patients (Fig. 2a) indicating that there are no genome-wide alterations in H3K4me3 methylation in PTSD when compared to controls. However, when individual genes were analyzed, we could see clear difference in H3K4me3 signals between controls and PTSD. Particularly, we could see higher H3K4me3 methylation around (within 3 kb) promoter region of *WNT10B*, *WNT10A*, *WNT7A*, *DVL1*, and *TCF7* (Fig. 2b). TCF7 (T cell factor 7) is a target gene of the Wnt signaling pathway activation which is predominantly expressed in T cells and plays a critical role in lymphoid cell development. These data suggested that the increased expression of these genes may be contributed by the presence of higher H3K4me3 signals in PTSD patients because the presence of H3K4me3 is associated with upregulation of the nearby genes due to chromatin accessibility made easier by the methylation of histone proteins (Wysocka et al. 2006).

### Knockdown of KDM5B demethylase results in increased WNT10B expression

Next, we determined if the presence of H3K4me3 around *WNT10B* promotor was controlling the expression of this gene. After knockdown of *KDM5B*, histone demethylase specific to H3K4me3 (Dey et al. 2008; Stalker and Wynder 2012), using siRNA (Fig. 2c), we found that the expression of *WNT10B* was indeed upregulated (Fig. 2d), thereby demonstrating that H3K4me3 was indeed involved in increasing the expression of *WNT10B*.

### Dysregulated miRNAs in PTSD target genes of the Wnt/β-catenin signaling pathway

**Fig. 3:**
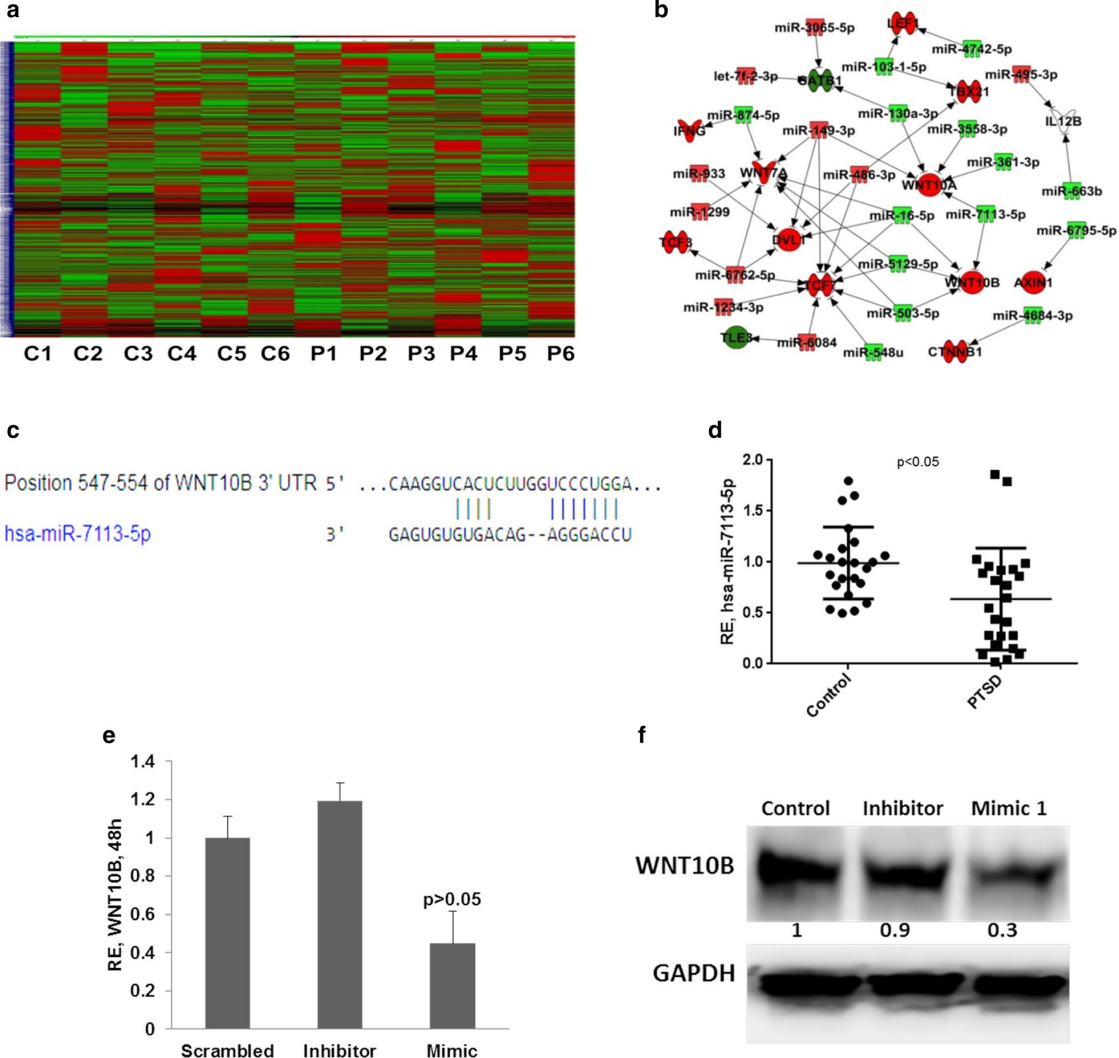
Micro-RNA expression analysis in PTSD patients. Microarray was performed on 6 controls and 6 PTSD patients each. **a** Heat map showing the normalized expression intensities of the miRNAs in controls and PTSD patients (C1–C6: Control, P1–P6: PTSD). **b** Dysregulated miRNAs were used for miRNA target gene identification in Ingenuity Pathway Analysis tool, Qiagen. The green color miRNAs are down regulated in PTSD and red color indicate those that are upregulated. **c** Nucleotide pairing schema for miR-7113-5p and WNT10B 3′UTR obtained from TargetScan. **d** RT-qPCR validation of hsa-miR-7113-5p expression in 24 controls and PTSD patients each. WNT10B transcript (**e**) and protein (**f**) levels in THP1 cells 48 h after transfection with hsa-miR-7113-5p mimics. The values in panel **f** indicate the normalized ratios of the different samples

We also performed microarray analysis to identify potential miRNAs that are dysregulated in PBMCs of PTSD patients (Fig. 3a). Based on fold change of miRNAs, we identified 68 miRNAs with at least ± 1.5 differences in PTSD patients when compared to controls, of which 35 were upregulated and the rest downregulated. We performed miRNA-gene interaction network analysis using Ingenuity Pathway Analysis (QIAGEN Inc., https://www.qiagenbioinformatics.com/products/ingenuity-pathway-analysis) (Kramer et al. 2014) with both sets of miRNAs obtained. Interestingly, the genes of Wnt/β-catenin signaling pathway were strongly predicted to be the targets of downregulated miRNAs indicating that upregulation of these genes in PTSD could also result from downregulation of these miRNAs (Fig. 3b). We further investigated the downregulated miRNAs and found that hsa-miR-7113-5p (among others) was strongly predicted to target *WNT10B* (Fig. 3c). This miRNA was downregulated in PTSD as per microarray analysis. To validate our microarray results, we performed qRT-PCR for hsa-miR-7113-5p in 24 samples from control and PTSD groups each and observed that it was significantly downregulated in PTSD patients when compared to controls (Fig. 3d). To show that the downregulation of hsa-miR-7113-5p can indeed lead to increased expression of *WNT10B*, we increased the amount of the miRNA in THP1 cells by introducing mimics of hsa-miR-7113-5p and assayed for the products of *WNT10B*. We observed significant reduction in the level of *WNT10B* mRNA (Fig. 3e) as well as protein (Fig. 3f) after treatment of cells with miRNA mimic for the indicated time compared to scrambled miRNA and miRNA-7113-5p-inhibitor treated groups. Together, these data showed that in PTSD, there is decreased expression of miRNAs such as hsa-miR-7113-5p that target WNT pathway, leading to its induction.

### Increase in WNT10B favors proinflammatory cytokine gene expression

**Fig. 4:**
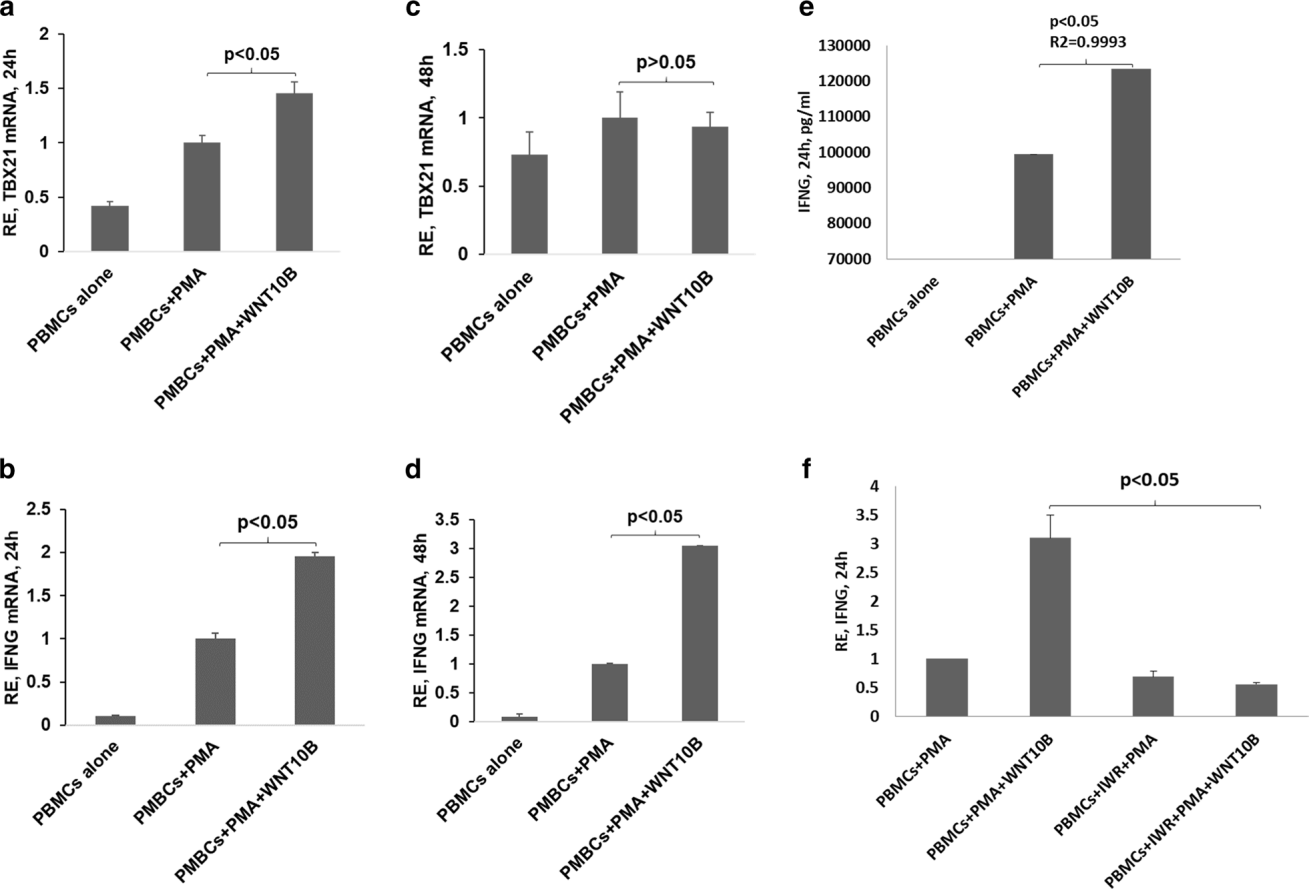
WNT10B-mediated effect on expression of proinflammatory genes. PBMCs from control individuals were cultured for indicated time with/without rh-WNT10B (200 ng/ml) after pre-activation of the cells with PMA (200 µM). **a**–**d** qRT-PCR results showing expression of Tbx-21 and IFNγ after WNT10B addition compared to activated PBMCs after the indicated time of culture. **e** IFNγ was measured in the culture supernatants after 24 h of WNT10B addition. **f** Inflammatory gene expression levels after blocking WNT signaling by IWR1 for the indicated time period. The first vertical bars represent controls against which the experiments were compared for fold change calculation. (IWR: IWR1, WNT signaling inhibitor)

Next, we investigated if upregulation of *WNT10B* could influence the expression level of inflammatory genes. To that end, we exposed Phorbol 12-myristate 13-acetate (PMA) stimulated PBMCs from normal controls to purified rh-WNT10B to mimic increased expression of *WNT10B*. The rh-WNT10B used in our study was similar to its use in previous studies by other groups (Thrasivoulou et al. 2013; Kilander et al. 2014; Jang et al. 2017). Addition of rh-WNT10B to PMA activated cells led to significant increase in the expression of pro-inflammatory genes such as *TBX21* and *IFN*γ at mRNA level at 24 h when compared to PMA activated PBMCs without rh-WNT10B (Fig. 4a, b). At 48 h, TBX21 in the rh-WNT10B added samples returned to level like activated PBMCs but IFNy was still significantly high (Fig. 4c, d). We also measured the protein level for *IFN*γ at 24 h and found it to be significantly increased in rh-WNT10B added group (Fig. 4e). It should be noted that addition of rh-WNT10B (200 and 500 ng/ml) alone to naïve PBMCs, administered either chronically or before stimulation with PMA, did not induce any inflammatory genes, including *STAT3*, *STAT4* or *IFN*γ (data not shown). These data suggested that the presence of rh-WNT10B during PBMC activation leads to enhanced induction of inflammatory cytokines.

### Inhibition of WNT signaling leads to lowered expression of inflammatory cytokine

Next, we tested if *WNT10B* signaling was directly involved in the upregulation of pro-inflammatory cytokine. To that end, WNT signaling was blocked by adding IWR-1 to the culture. Upon blocking of WNT signaling, the expression of *IFN*γ was significantly downregulated compared to cells activated with PMA only. Furthermore, even the addition of *WNT10B* did not lead to increased expression of *IFN*γ and other proinflammatory genes (Fig. 4f) even after activation of PBMCs with PMA.

### WNT pathway gene expression in replication study groups

**Fig. 5:**
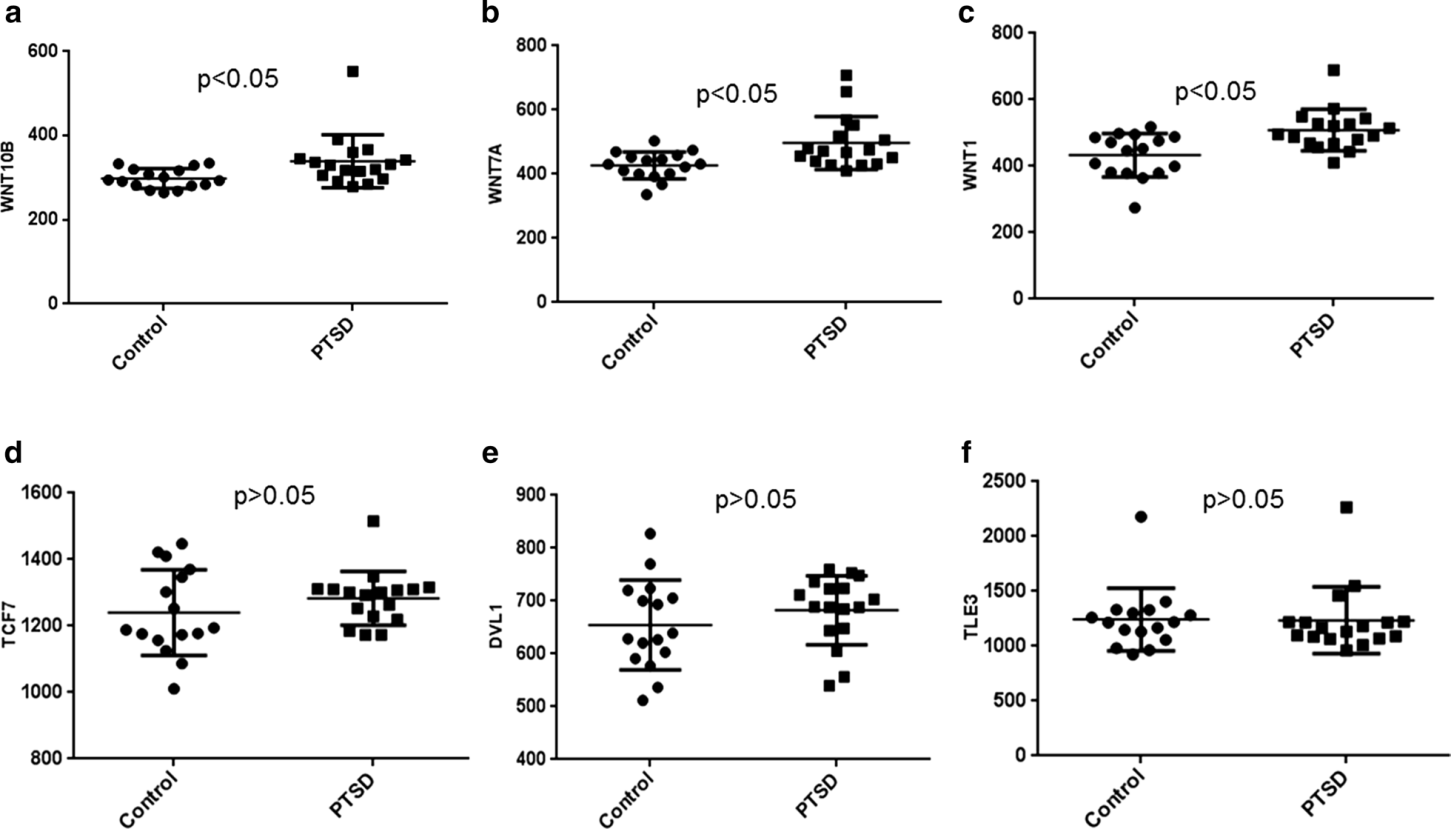
WNT pathway gene expression analysis from GEO Dataset. We compared the expression levels of WNT pathway genes in a GEO dataset (GSE860, Dataset 2), with data obtained from PBMCs of PTSD and controls by microarray, to confirm the observations in our dataset obtained from samples we collected. **a**–**f** WNT pathway genes expression levels in Dataset 2. **a**–**c** corresponds to different WNT ligands upregulated in PTSD. One symbol represents an individual. The expression level corresponds to the values obtained from the analysis performed on GEO2R in the GEO dataset link. The *p* values were obtained by performing Student’s *t* test on the values obtained from the link

As mentioned earlier, we further confirmed our results by comparing data from an additional dataset (Dataset 2, GSE860) available in NCBI’s repository. Analyses with this dataset confirmed significant upregulation of *WNT10B*, *WNT7A* and *WNT1* (Fig. 5a–c), corroborating our earlier observations. Furthermore, *TCF7*, *DVL1* and *TLE3* also showed similar trend in their expression pattern even though the difference was statistically not significant (Fig. 5d–f).

## Discussion

PTSD is considered a serious psychiatric disorder with chronic inflammation in the patients (Daskalakis et al. 2016; Speer et al. 2018). T cells are believed to be the main players in regulating inflammation. Increased activation state of CD4+ T cells and the increased numbers of CD4+ and CD8+ T cells in PTSD patients were reported by our laboratory, previously (Zhou et al. 2014; Bam et al. 2016a, b). However, there is very limited understanding of how the inflammation is initiated and sustained to become chronic after an exposure to traumatic event. These findings suggest that a cellular signaling pathway controlling cell differentiation and proliferation is probably dysregulated in PTSD. In the current study, using RNA-seq screening analysis, we found that Wnt/β-catenin signaling pathway was upregulated in PTSD, which is known to play a critical role in cell differentiation, growth, proliferation, survival, and immune cell function (Logan and Nusse 2004; Nusse 2005; Houschyar et al. 2018; Staal and Arens 2016; Kling and Blumenthal 2017; Gilbert 2010). Specifically, we observed an increase in *WNT10B* expression (including others like *WNT10A* and *WNT7A*) in PTSD. Our further observations after analyzing data from two larger datasets clearly confirms this observation and suggests that WNT signaling pathway genes are indeed dysregulated in PTSD patients. Furthermore, analyzing transcriptomic data from hippocampus of mice exposed to stress, Bian et al. identified that WNT signaling pathway genes (CYP1A2, SYT1 and NLGN1) were dysregulated in these mice indicating that this signaling pathway could be involved in PTSD (Bian et al. 2019). This observation is very interesting because it further suggests that WNT signaling could be playing an important role in PTSD pathophysiology. Dysregulation of the Wnt/β-catenin pathway has been reported recently in several neurodegenerative diseases (Al-Harthi 2012), including Alzheimer’s disease (Ferrari et al. 2014), Parkinson’s disease (Grand et al. 2015), and multiple sclerosis (Giacoppo et al. 2017). Thus, it is interesting that our studies detected for the first time dysregulated Wnt signaling pathway in PBMCs of PTSD patients who often develop both immunological and psychiatric disorders. Wnt signaling pathway has been shown to play a role in T cell development (Staal and Arens 2016; Roozen et al. 2012). However, its role in mature T cell function is less clear. Interestingly some recent studies suggested that Wnt signaling in T cells was elevated which promoted expression of RORγt and consequently inflammatory Th17 cells (Keerthivasan et al. 2014). These data are consistent with the current study in which we found a positive correlation between enhanced Wnt signaling with proinflammatory IFNγ induction. During an inflammatory reaction, there is an elevated production of cytokines produced by different types of immune cells and in PTSD it has been shown that expression of pro-inflammatory cytokines like IFNγ and IL-12 are elevated (Zhou et al. 2014; Bam et al. 2016a). The elevated production of cytokines requires activated state of cells that produce them. We observed that upon increasing the WNT10B signaling after addition of rh-WNT10B, the production of proinflammatory cytokines was further enhanced in pre-activated PBMCs. Previous studies have reported the use of rh-WNT10B to study WNT signaling in context to regulation of TNF-α (Jang et al. 2017) under inflammatory settings. In another study, Kilander et al. (2014) used rh-WNT10B and other WNT ligands to study the mobility of FZD6 receptor on the membrane. Furthermore, it was reported that Wnt signaling pathway is impaired during inflammatory condition leading to increased pro-inflammatory cytokine synthesis (Jang et al. 2017; Houschyar et al. 2018; Keerthivasan et al. 2014). Thus, our observations from use of rh-WNT10B further suggest that activated T cells in PTSD may be driven towards proinflammatory phenotype by enhanced Wnt signaling. Moreover, by blocking WNT signaling, we further confirmed that indeed the enhancement of cytokine production was because of increased WNT10B expression.

Epigenetic dysregulation of Wnt/β-catenin signaling genes has been well characterized in cancer. In a review article, Wils and Bijlsma (2018) has listed and described the regulation of various WNT ligands and WNT signaling pathway downstream genes by epigenetic mechanisms in different cancer types. For example, WNT10B is downregulated in colorectal cancers as a result of promoter hypermethylation (Yoshikawa et al. 2007) and, on the contrary, WNT2 is upregulated due to histone modifications (Jung et al. 2015). However, epigenetic regulation of WNT signaling pathways in immune cells and their functioning in PTSD is not clearly understood. To that end, we first investigated the role of histone methylation and found that *WNT10B* was regulated, at least in part, by H3K4me3, a histone mark that is responsible for upregulation of a gene harboring this mark. PTSD patients had higher H3K4me3 signal around the promotor region of *WNT10B*, clearly indicating that this histone modification could be leading to upregulated expression of WNT10B in PTSD. This was further confirmed by our observation that manipulation of H3K4me3 by siRNA knock down of KDM5B led to upregulation of WNT10B. Previously, we have shown that increased H3K4me3 around IFNγ was responsible for the upregulation of the gene in PTSD patients (Bam et al. 2016a). In line with this, our present observation also suggests that the increased H3K4me3 around *WNT10B* may contribute to its higher expression in PTSD. When ChIPseq data from our previous publication (Bam et al. 2016b) was compared with the present results, we observed that there were far lesser H3K4me3 marks in our present analyses compared to the previous ones. We believe that this is a result of the vast difference in the number of cells used for the ChIPseq (> 10million cells in the previous study and only about 40,000 cells in the present study).

With regards to miRNA–mRNA interaction, it is well established that interaction of miRNA with the target mRNA leads to degradation of the mRNA to the extent of > 66–90% in the mammalian system (Eichhorn et al. 2014). We were interested in understanding the probable involvement of miRNAs as well in the regulation of *WNT10B* expression in PTSD as we have previously reported significant downregulation of miRNAs in PTSD patients and showed that IFNγ, IL-12 and several other genes of the immune system, are regulated by miRNAs whose expression is decreased in PTSD (Zhou et al. 2014; Bam et al. 2016a, b, 2017). As indicated by the mimic transfection assays, we confirmed that downregulation of miRNA hsa-miR-7113-5p was indeed responsible for the upregulated state of *WNT10B*. Because our previous reports have shown that PTSD is associated with several downregulated miRNAs that ultimately were responsible for the induction of *IFN*γ, *TBX21* and *IL12* (Zhou et al. 2014; Bam et al. 2016b), the present data involving hsa-miR-7113-5p suggests that this mechanism may also play a role ultimately affecting the expression of proinflammatory genes by inducing *WNT10B*.

**Fig. 6:**
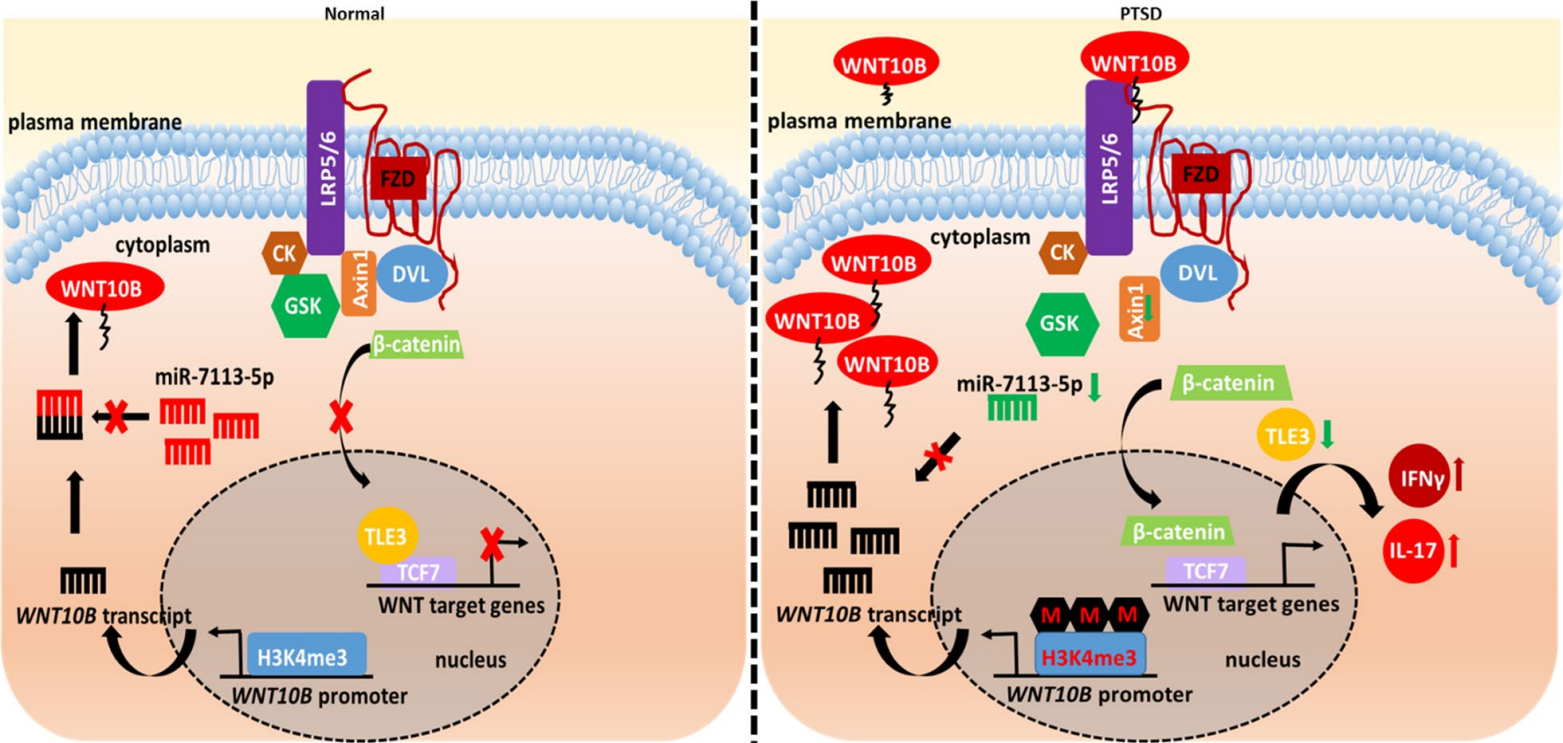
Schematic showing our working hypothesis of WNT10B elevation leading to increased inflammatory gene expression because of histone modification and miRNA expression alteration in PTSD

It is still unclear as to what triggers chronic inflammation in PTSD. Importantly, it is not known whether a traumatic event causes the initiation of the inflammatory condition leading to cognitive dysfunction seen in PTSD or whether the inflammation is independent of exposure to trauma. Some authors have suggested that in neuropsychiatric disorders, there is significant crosstalk between inflammation and the clinical symptoms. For example, depression facilitates inflammatory reactions and inflammation in turn promotes depression and other neuropsychiatric disorders (Bauer and Teixeira 2018). Whichever occurs first, the question is what triggers the immune system in PTSD? It has been suggested that the triggers could include changes in neuroendocrine regulation, metabolism, diet/microbiota, and negative health behaviors (Bauer and Teixeira 2018). Based on studies from our lab, we have hypothesized that exposure to trauma may trigger epigenetic changes leading to alterations in the expression of microRNA, DNA methylation and/or histone modifications that target genes involved in immune system regulation, thereby promoting inflammation (Zhou et al. 2014; Bam et al. 2016a, b, 2017). Thus, based on our current study, we propose that, as depicted in Fig. 6, exposure to trauma may trigger epigenetic changes such as higher H3K4me3 methylation and dysregulated miRNA expression in PTSD which leads to induction of WNT10B that further enhances the inflammatory response.

There is significant evidence to suggest that patients with PTSD are also at greater risk for diseases that affect multiple organ systems, including cancer, arthritis, digestive disease, and cardiovascular disease (Brudey et al. 2015). Interestingly, chronic inflammation is also the underlying cause of such clinical disorders. Thus, further studies are necessary to address the role of chronic inflammation in comorbidity associated with PTSD. Another important area would be to look into the effect of gender in the development of PTSD involving WNT10B signaling dysregulation. Moreover, considering the fundamental role of WNT signaling pathway and the recent reports showing its involvement in cancers and other diseases, a detailed study of this signaling pathway in the regulation of inflammatory mechanisms is warranted to open a new research avenue in PTSD. Inasmuch as PTSD is a complex disorder, it will be important to elucidate whether the dysregulated role of WNT signaling in PTSD predisposes these patients to other clinical disorders and whether trauma experienced by PTSD patients leads to epigenetic changes which in turn lead to a defect in WNT signaling. In the current study, although the findings were original and novel, one of the major limitations was the small number of samples. Thus, we believe that similar studies with a larger sample size will shed more light and further corroborate the data presented in this study.

## Conclusions

In summary, our data demonstrate for the first time that Wnt signaling pathway is upregulated in PBMCs of PTSD patients resulting from epigenetic changes involving microRNA dysregulation and histone modifications, which in turn may promote the inflammatory phenotype in such cells. Therefore, we conclude that dysregulated WNT10B may play a key role in enhancing inflammation in PTSD as a result of dysregulated epigenetic mechanisms.

## Data Availability

The datasets used in this study are available from the corresponding author on reasonable request.

## Abbreviations

PTSD: Posttraumatic stress disorder
WNT: Wingless-type MMTV integration site
IFNγ: Interferon gamma
IL-12: Interleukin 12
TBX21: T-box transcription factor 21
H3K4me3: Tri-methylation at the 4th lysine residue of the histone H3 protein
KDM5B: Lysine demethylase 5B
TCF7: Transcription factor 7
DVL1: Dishevelled 1
TLE3: Transducin-like enhancer of split 3
CTNNB1: Catenin beta-1
PBMC: Peripheral blood mononuclear cells
PMA: Phorbol 12-myristate 13-acetate
FPKM: Fragments per Kilobase of transcript per Million mapped reads
IGB: Integrated genome browser
miRNA: Micro RNA
ChIPseq: Chromatin immunoprecipitation sequencing
RNAseq: RNA sequencing
